# 

**Published:** 1884-04-20

**Authors:** 


					﻿^®F*Many subscribers are in arrears for subscription. Friends,
f lease settle at once, and oblige	R. C. Word,
Managing Editor,
See the interesting advertisement of J. S. Pemberton & Co. in this issue.
See the new advertisement of that nice preparation of T. B. Wheeler, M.D.
Harter’s Iron Tonic.—See the advertisement and special notice in this
issue of the above excellent preparation.
The American Medical Association mests in Washington, D. C.,
on the 6th of May instead of the ist, as stated in our last.
The Missouri State Medical Association is appointed for May
5th, 1884, at Sedalia.
The Arkansas Medical Society meets in Little Rock on Wednesday,
April the 30th.
The Association of American Medical Editors will convene in
Washington on May the 5th, in Medical Hall, S. E. corner of Sixth and F
streets, at eight P. M. Dr. L Connor will deliver the Annual Address. Sub-
ject: “The American Medical Journal of the Future.”
American, Medical Association.—Arrangements for reduced fare to
the Association are being made with all railroads leading to Washington city.
Parties desiring information are referred to Dr. D. W. Prentiss, of Washing-
ton, D. C.
The Prize on Anatomy presented by Prof. W. P. Nicolson at the late
Commencement of the Southern Medical College was, in some unaccountable
way, overlooked in the proceedings of that interesting occasion as published in
the last copy of our Journal. The recipient of this honor in this, the most dif-
ficult and important of all the text-books, was D. W. S. Bradley, of Adairs-
ville, Qa., who stood very high, also, in other branches, and is a young man of
fine promise.
RECEIPTED.
1884.—Drs. L. W. Coleman; J. H. Boggan; J. J. Groover; W. E. Courson; John H.
Owings; Louis Hadden; R. 8. Powell to January, ’85; T. L. Quillian; B. F. Duke;
1883.—T. T. Seals; James R. Wiley; Thomas Sayers; Evan Williams; J. I. Moon;
8. A. Moore; K. K. Small; L. N. Raines.
				

